# The radiosensitization effects of Endostar on human lung squamous cancer cells H-520

**DOI:** 10.1186/1475-2867-10-17

**Published:** 2010-05-24

**Authors:** Zhen Y You, Yong Zhao, Feng Liu, Ying D Zhang, Jun J Wang

**Affiliations:** 1 Cancer Center, Department of Radiation Oncology, Peking University Third Hospital, Beijing 100191, China; 2 Transplantation Biology Research Division, State Key Laboratory of Biomembrane and Membrane Biotechnology, Institute of Zoology, Chinese Academy of Sciences, Beijing 100101, China

## Abstract

**Background:**

The present study mainly aimed to investigate the direct effects of Endostar (ES) on the proliferation and radiosensitivity of human lung squamous cancer cell line H-520.

**Results:**

ES significantly inhibited H-520 cell proliferation in a time- and dose-dependent manner. According to the colony-forming assays, ES could increase the H-520 cell radiosensitivity. ES induced cell apoptosis, the apoptosis rate increased with the raise of ES concentration. Irradiation induced significantly higher apoptosis rate in ES-treated H-520 cells than non-treated H-520 cells. ES induced cell cycle distribution and G_0_/G_1 _arrest in H-520 cells, whereas irradiation induced G_2_/M arrest. The phospho-p38-MAPK and p-Akt protein levels were decreased in H-520 cells after ES treatment. Furthermore, activated caspase protein level increased and Bcl-2 protein levels decreased after treatment with ES and irradiation.

**Conclusion:**

ES significantly enhanced the sensitivity of H-520 cells to irradiation by inhibition of cellular proliferation, promotion of cell apoptosis and redistribution of cell cycle, possibly via deactivation of Akt pathway. The present study supports the possibility to use the combination of ES and ionizing irradiation to treat patients with lung squamous cell cancer in clinics.

## Background

About 40% of patients with stage III or IV non-small cell lung carcinoma (NSCLC) cannot be resected at present [1]. Radiotherapy and chemotherapy are still the major treatment in such patients and significantly improves the survival of unresectable patients [1-3]. Nevertheless, long-term survival remains poor and mortality is high. Anti- angiogenesis therapy that interfered with cancer angiogenesis may improve lung cancer patient survival by enhancing radiation and chemotherapy efficiency without increasing treatment-related adverse effects [4-7].

Endostatin (ED) is a 22 kDa polypeptide derived from the C-terminal fragment of type XVIII collogen. Both recombinant human and murine ED have been reported to inhibit endothelial cell proliferation but not smooth muscle cells or fibroblast proliferation in vitro, which suggested that the anti-proliferation effect was endothelial-specific [8,9]. The anti-angiogenic activity of ED was a complicated process resulting in the inhibition of endothelial cell adhesion, migration and proliferation as well as the induction of apoptosis [10,11]. It has been shown that ED bind specifically to the cell surface receptor on the endothelial cells and the complex of ligand-receptor was internalized into the cells [12-14]. The integrin α_ν_, α_5_, β_1 _and cell surface glypicans and nucleolin have been identified as receptors interact with ED [14-16]. Recently, the anti-tumor effects of ED have been reported. Wilson *et al *reported that ED inhibited migration and invasion of head and neck squamous cell carcinoma cells [17]. Cui *et al *reported that ED directly modulated lung cancer cell function in vitro [18]. Dkhissi *et al *demonstrated that ED exhibited a direct anti-tumor effect in addition to its anti-angiogenic activity in colon cancer cells [11]. All these results suggested that ED could interact with not only endothelial cells but also cancer cells. However, it was not reported whether ED directly modulates NSCLC cell function such as proliferation, apoptosis and cell cycle distribution or whether it has the ability to enhance radiosensitization activity in NSCLC cells.

Endostar (ES) is a novel recombinant human ED which expressed and purified in *E. coli*, and was approved by the State Food and Drug Administration (SFDA) for the treatment of NSCLC in 2005 [19]. ES is traditional ED with an additional nine-amino acid sequence at the N-terminal of the protein and a six-histidine tag which could be chelated with metal ions with a relatively high affinity. As a result, the purification is simplified and the stability of the protein was remarkably improved [20]. In the present study, we evaluated the direct radiosensitive effects of ES on human lung squamous carcinoma cells H-520 in vitro and also explored its mechanism of radiosensitization.

## Materials and methods

### Cell lines and cell culture

The human lung squamous cancer cell line H-520 was purchased from the Institute of Basic Medical Sciences Chinese Academy of Medical Sciences and cultured in DMEM supplemented with 100 IU/mL penicillin, 100 mg/mL streptomycin, 4 mM glutamine and 10% heat-inactivated fetal bovine serum (Hangzhou Sijiqing Biological Engineering Materials Company, China) in a humidified atmosphere of 95% air and 5% CO_2 _at 37°C. ES-treated H-520 cells were obtained by culturing cells with 200 μg/mL ES (expressed and purified in E. coli. Simcere Pharmaceutical Research Co., Ltd) for 24 h before irradiation. Phospho-P38 mitogen-activated protein kinases (MAPK) mAb (Alexa Fluor) was provided by Cell Signaling Technology, US.

For all in vitro experiments, cells were released from flasks using phosphate- buffered saline containing 0.01% trypsin and 0.20 mmol/L EDTA, and 1 × 10^5 ^cells were plated onto 25 cm^2 ^culture flasks one day before drug treatment. Cultures were between 50% and 70% confluence at the time of harvest.

### Cell growth assay

Cells were maintained as mono-layer cultures in DMEM with 10% FBS and antibiotics (100 U/ml of penicillin and l00 mg/ml streptomycin). The cells were incubated in 5% CO_2 _atmosphere. The studies were completed with 3 × l0^3 ^cells/well plated into 96-well flat bottom microplates (Costar), treated with ES of desired concentration when cells began to grow exponentially. After incubation for 24-96 h, 20 μL of 3-(4,5-diethyl-2-thiazolyl)-2,5-diphenyl tetrazolium bromide (MTT 5 mg/mL) was added to each well, and the cells were further incubated at 37°C for 4 h. The medium was then removed and 200 μL of DMSO was added to dissolve the reduced formazan product. The plate was then read on a microplate reader (Bio-RAD, model 550) at 590 nm.

### Ionizing radiation treatment

Exponentially growing H-520 cells were irradiated using ^60^Co γ ray source at 1.953 Gy/min dose rate. Radiation was performed in the Radiation Department of Peking University.

### Clonogenic assay

H-520 cells of the control and ES groups were exposed to different radiation doses (0,1, 2, 4, 6, 8 and 10 Gy) and incubated for 21 d, then cells were fixed with methanol and stained with Giemsa. Cell colonies which contained more than 50 cells were manually counted. Radiation survival data from ES-treated cells were corrected for plating efficiency (PE) using an unirradiated plate treated with ES under the same conditions. The PE and survival fraction (SF) were calculated as follows: PE = (colony number/inoculating cell number) × 100%. SF = PE (tested group)/PE (0Gy group) × 100%. A dose-survival curve was obtained for each experiment and used for calculating the radiobiological parameters. Three replicates were set at each radiation dose. The cell-survival curve was fitted using Origin 7.5 software according to the multi-target single-hit model after normalizing for the cytotoxicity induced by Endostar alone. The equation of SF = 1-(1-e^-D/D0^)^N ^was applied to calculate the cellular radiosensitivity (mean lethal dose, D_0_), the capacity for sublethal damage repair (quasithreshold dose, Dq), and the extrapolation number (N). Those values were used to calculate the SF after irradiation at a dose of 2Gy (SF_2_) and the sensitization enhancement ratio (SER).

### Apoptosis assay

Two methods were used to analysis apoptosis induced by ES. Morphological changes were determined by Hoechst33258 staining. Single cell suspensions in PBS were stained with 10 μg/mL Hoechst33258. A drop of the stained cell suspension was placed on a microscope slide. Cells were visualized under a fluorescence microscope with a blue filter. Apoptotic cells were defined as cells showing cytoplasmic and nuclear shrinkage and chromatin condensation or fragmentation morphologically. Annexin V-FITC and propidium iodide (PI) double staining were used for evaluation of cellular apoptosis rate. The H-520 cells were treated with 200 μg/mL ES for 24 h and then irradiated with different doses of 0, 2, 4 and 8Gy. After a 72 h post- irradiation incubation period in 200 μg/mL, all cells were collected, stained with annexin V-FITC for 30 min at 4°C, then stained with PI and analyzed by flow cytometry (FCM) immediately.

### Activated caspase proteins assayed by FCM

5 × 10^5 ^H-520 cells were harvested after treatment with irradiation for 24 h. Then, washed once in 1 ml PBS, and re-suspended in 200 μl staining solution containing FITC-VAD-fmk (CaspACE, Promega). After incubation for 20 min at 30°C, cells were washed in 1 ml PBS and re-suspended in 200 μl PBS [21]. Samples were analyzed by FACScan using Coulter EPICS and ModFit software.

### Analysis of cell cycle by FCM

H-520 cells from the control and ES groups were treated with irradiation (0 and 4Gy) and 8, 24, and 48 h later, cells were trypsinized, counted, washed, and re-suspended from all flasks. Cells were then fixed by dropwise addition of 70% ice-cold ethanol and stored at 4°C until the day of analysis. Cells were then washed in phosphate-buffered saline, and re-suspended in PI solution at 50 μg/mL and analyzed by FCM using Coulter EPICS and ModFit software (Verity Software House, Topsham, MN).

### Phospho-P38-MAPK quantification by FCM

Bcl-2 and phosphor-p38-MAPK were quantified in control and ES-treated H-520 cells at 24 h after irradiation. Cells were stained with p38-MAPK mAb (Alexa Fluor) or Bcl-2 mAb (Beijing Zhongshan Golden Bridge Biotechnology Co., Ltd) and analyzed by FACScan using Coulter EPICS and ModFit software.

### Western blotting analysis

H-520 cells from the control and ES groups were treated with irradiation (0 and 4Gy), harvested and total cell lysates were resolved on 10% SDS-PAGE, and processed according to standard protocols. The antibodies (Abs) used for western blotting included: monoclonal anti-β Actin (Sigma, USA), monoclonal antibody against phosphor-Akt (Beijing Zhongshan Golden Bridge Biotechnology Co., Ltd). Optimal dilutions of primary Abs were 1:1000 to 1:5000. The secondary Abs anti-rabbit were conjugated to horseradish peroxidase (dilution 1: 5000 to 1: 10000); signals were detected using the ECL system.

### Statistics

Results are presented as mean ± SE of at least three experiments. Student's t test was used to assess the statistical significance of differences. A significance level threshold of *p *< 0.05 was used in this study.

## Results

### The effects of ES on cell proliferation

**Figure 1:**
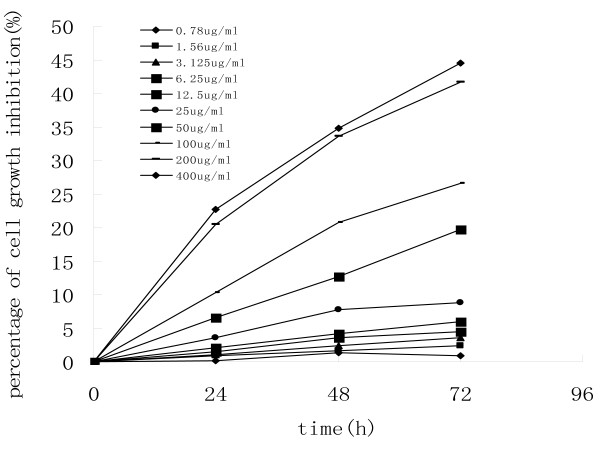
**MTT assay was used to evaluate the effect of ES on the growth of H-520 cells**. The exponentially growing H-520 cells were seeded in 96-well plates and treated with increasing concentrations of ES for 24, 48 and 72 h. The results are presented as the percentage of the decreased values from the untreated cells. One representative of two identical experiments was shown.

The effect of ES on the proliferation of human lung squamous cancer cells H-520 was first determined using MTT assay. As shown in Fig. 1, ES significantly inhibited the proliferation of H520 cells in a dose- and time-dependent manner, especially when the concentrations of ES were between 25 and 200 μg/ml. Based on the present results, we chose 200 μg/mL ES as an appropriate concentration for the following experiments.

### Radiosensitization of ES in H-520 cells

**Figure 2:**
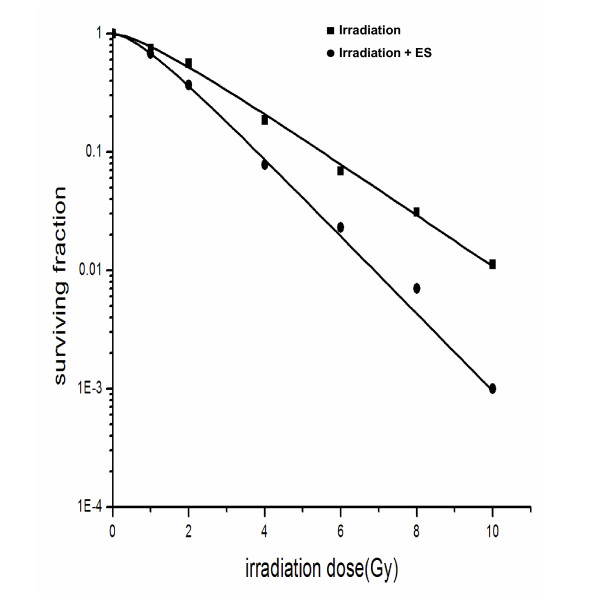
**Clonogenic survival of H-520 cells after different treatments including irradiation with or without ES**. H-520 cells were treated with 200 μg/mL ES for 24 h and then washed. These ES-treated or control H520 cells received 0-10Gy radiation and cloning formation was detected by 21 days after irradiation. Data were a summary of three experiments.

In the coming assays, we treated H-520 cells with ES in a dose of 200 μg/mL for 24 h before those cells received different doses of irradiation. The cell survival was detected by a clonogenic assay at 21d after irradiation. As shown in Fig. 2, survival fraction of H-520 cells at 2Gy (SF_2_) was 0.56 vs. 0.37 and mean inactivation dose (D_0_) was 2Gy vs. 1.33Gy in the absence and presence of ES pre-treatment respectively. The SER of ES in H-520 cells was 1.51. The other radiobiological parameters of H-520 cells were: Dq = 0.41 Gy, N = 1.60 and D_10 _= 4.60Gy, whereas these parameters of ES-treated H-520 cells were: Dq = 0.34Gy, N = 1.79 and D_10 _= 3.05Gy, respectively. These data indicated that ES had efficient radiosensitization effects on H-520 cells in vitro.

### Cell death of H-520 cells induced by ES and irradiation

**Figure 3:**
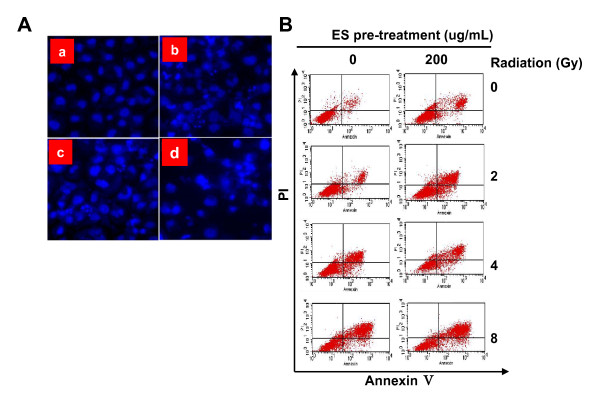
**ES treatment significantly enhanced radiation-induced cell death in H-520 cells**. **A**) Cell apoptosis observed by Hoechst 33258 staining (×400). H-520 cells treated with medium alone (**a**), 200 μg/mL ES (**b**), irradiation (**c**), and their combination (**d**) for 72 h. **B**) Cell death after different treatments was determined by a flow cytometry. The early apoptosis rate of ES-treated H-520 cells was significantly higher than those of un-treated H-520 cells at the radiation dosage of 4Gy. Higher total apoptosis rate of the ES-treated H-520 cells was observed at every radiation dose included in this study. One representative of three identical experiments was shown.

To evaluate the induction of apoptosis after the combination of ES and irradiation, H-520 cells were treated with 200 μg/mL ES for 24 h immediately before irradiation. Apoptosis cell death was determined by fluorescence microscopy using cells stained with Hoechst 33258. H-520 cells showed typical morphologic changes of apoptosis as cytoplasmic and nuclear shrinkage and chromatin condensation or fragmentation morphology (Fig. 3A).

The amount of apoptotic cells was determined by annexin V-FITC and PI double staining. As expected, more apoptotic cells were observed when the radiation doses increased (Fig. 3B and Table 1). Radiation induced significantly higher cell death in ES-pretreated H-520 cells than those in control H-520 cells (P < 0.05 or P < 0.01, Table 1).

**Table 1:** Effects of ES and irradiation on apoptosis in H-520 cells(%, ± s)

Irradiation dose(Gy)	H-520 cells		ES-treated H-520 cells	
		
	Early apoptotic rate	Total apoptotic rate	Early apototic rate	Total apoptotic rate
0	0.57 ± 0.03	4.27 ± 0.29	4.59 ± 0.48^a^	22.38 ± 1.61^b^
2	3.99 ± 0.48	14.3 ± 1.15	9.54 ± 0.29^c^	35.01 ± 1.16^d^
4	6.8 ± 0.26	28.49 ± 1.58	12.08 ± 0.25^e^	46.83 ± 2.06^f^
8	7.46 ± 0.24	54.79 ± 1.89	8.68 ± 0.68^g^	64.08 ± 4.28^h^

Compared to the control H-520 cells irradiated with the same dose,*t *= 14.40, ^a^*P *< 0.01; *t*= 19.17, ^b^*P *< 0.01; *t *= 17.15, ^c^*P *< 0.01; *t *= 17.79, ^d^*P *< 0.01; *t *= 25.64, ^e^*P *< 0.01; *t *= 25.64, ^f^*P *< 0.01; *t *= 2.93, ^g^*P *< 0.05; *t *= 3.44, ^h^*P *< 0.05.

### Effects of ES and irradiation on cell cycle progression of H-520 cells

**Figure 4:**
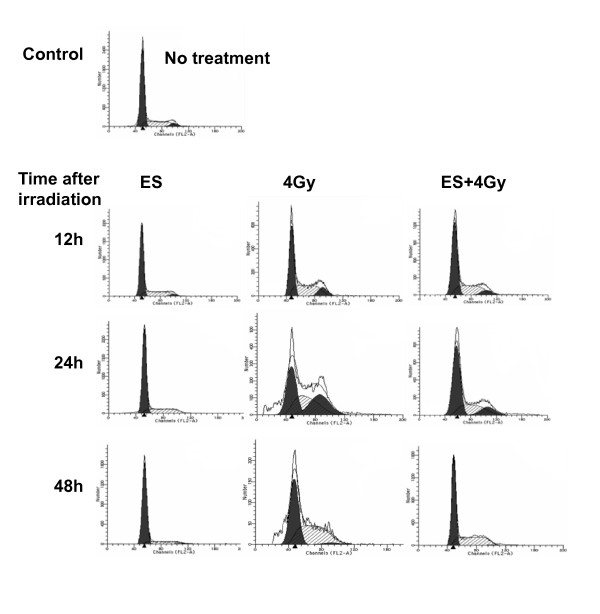
**Cell cycle distribution of H-520 cells after treatment with ES and/or 4Gy irradiation from 12 to 48 h in H-520 cells**. One representative of two independent experiments with similar results was shown.

H-520 cells were treated with ES (200 μg/mL) for 12 to 48 h, the G_0_/G_1 _peak was increased and the G_2_/M peak decreased in a time-dependent style. There was no significant change in the S-phase after treated with ES for 12 h, but a marked decrease was observed from 24 to 48 h after treatment with ES (Fig. 4 and Table 2). Exposed to 4Gy irradiation for 12-48 h, the G_0_/G_1 _peak decreased, But the G_2_/M peak increased after irradiation during 12 to 24 h and decreased from 24 to 48 h. However, the combination of ES and irradiation induced significantly decreased G_0_/G_1 _and G_2_/M phase cells but increased cells in S phase compared with control cells (Table 2).

**Table 2:** Cell cycle distribution in H-520 cell line after treatment with ES and irradiation(%,  ± s)

Group	Treatment	G_0_/G_1_	S	G_2_/M
0 h	No treatment	66.15 ± 1.01	28.18 ± 1.23	5.66 ± 0.22
12 h	ES	66.03 ± 1.46	30.66 ± 2.55	3.31 ± 1.10^a^
	4Gy	47.27 ± 1.25^b^	41.1 ± 1.67^c^	11.63 ± 1.65^d^
	ES+4Gy	58.62 ± 3.84^e^	34.32 ± 2.88^f^	7.06 ± 0.97
24 h	ES	72.36 ± 2.14^g^	27.03 ± 1.71	0.61 ± 0.54^h^
	4Gy	35.79 ± 1.43^i^	33.94 ± 1.79^j^	30.23 ± 0.43^k^
	ES+4Gy	57.34 ± 2.26^l^	29.11 ± 2.39	13.55 ± 0.78^m^
48 h	ES	82.72 ± 3.32^n^	15.06 ± 3.13^o^	2.22 ± 0.94^p^
	4Gy	48.85 ± 3.28^q^	48.12 ± 2.10^r^	3.03 ± 1.35^s^
	ES+4Gy	57.78 ± 1.46^t^	41.44 ± 1.08^u^	0.78 ± 0.39^v^

Compared with 0 h group, t = 3.63, ^a^P < 0.05; t = 20.36,^b^P < 0.01; t = -10.79,^c^P < 0.01; t = -6.22, ^d^P < 0.01; t = 3.29, ^e^P < 0.05; t = -3.40, ^f^P < 0.05; t = -4.54, ^g^P < 0.05; t = 14.94, ^h^P < 0.01; t = 30.12, ^i^P < 0.01; t = -4.60, ^j^P < 0.05; t = -87.69, ^k^P < 0.01; t = 6.17, ^l^P < 0.01; t = -16.92, ^m^P < 0.01; t = -8.28, ^n^P < 0.01; t = 6.76, ^o^P < 0.01; t = 6.15, ^p^P < 0.01; t = 8.75, ^q^P < 0.01; t = -14.18, ^r^P < 0.01; t = 3.33, ^s^P < 0.05; t = 8.16, ^t^P < 0.01; t = -14.08, ^u^P < 0.01; t = 18.82,^v^P < 0.01.

### Effects of ES and irradiation on proteins involved in apoptosis

**Figure 5:**
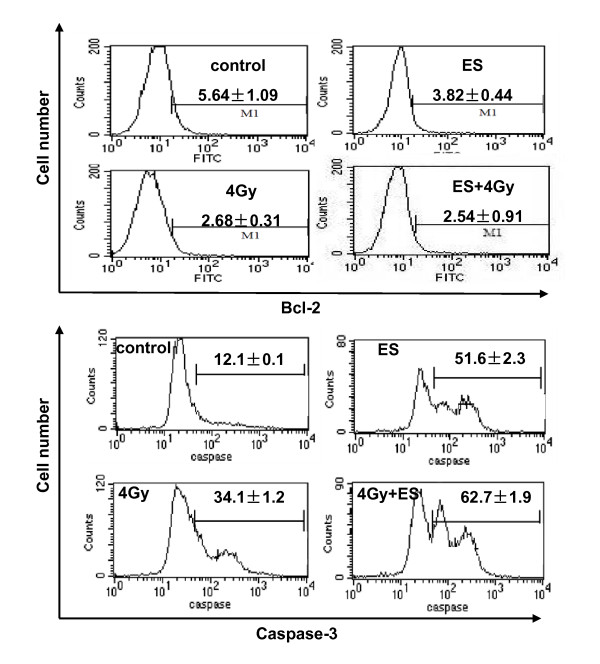
**Bcl-2 and activated caspase protein level in H-520 cells after treatment with ES and 4Gy irradiation were detected by a flow cytometry**. **A**) Decreased Bcl-2 expression in ES and radiation-treated H-520 cells. **B**) Increased activated caspase level in H-520 cells induced by ES and irradiation. Data were presented as mean ± SD(N = 4). One representative of four identical experiments was shown.

Proteins involved in apoptosis including Bcl-2 and activated caspase which were tested by flow cytometry. The Bcl-2 expression was decreased in H-520 cells after the treatment with irradiation or ES, but less expression of Bcl-2 in H-520 cells was observed after the treatment with both ES and irradiation (Fig. 5A). On the other hand, the activated caspase level was up-regulated in irradiation- or ES- treated H-520 cells compared with control H-520 cells. The combination of ES and irradiation induced significantly higher activated caspase level in H-520 cells compared with the control H-520 cells (Fig. 5B).

### Expression of phospho-p38-MAPK and p-Akt in H-520 cells after ES and irradiation treatment

**Figure 6:**
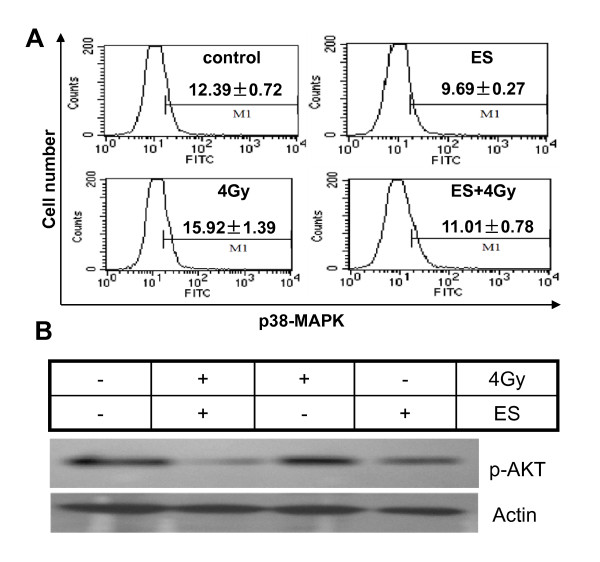
**Phosphorylation level of p38-MAPK and Akt in H-520 cells after irradiation and ES treatment**. **A**) ES combined with 4Gy irradiation significantly inhibits p38-MAPK phosphorylation. **B**) Decreased p-Akt in H-520 cells caused by ES treatment. Protein extracts of cells treated with 200 μg/mL ES, 4Gy irradiation, and in combination (ES+4Gy) at 24 h following irradiation. One representative of three independent experiments was shown.

ES influenced signaling pathway, we examined the expression of the phosphorylation status of Akt by Western blotting and p38-MAPK by FCM, which mainly associated with mitogenicity and cell proliferation. As shown in Fig. 6A and Table 3, ES could significantly inhibit the phosphorylation of p38-MAPK in H-520 cells after treatment with irradiation or not. However, the level of phosphor-p38-MAPK in cells treated with irradiation and ES had no observed change. Western blotting analysis showed significant decrease of the phosphor-Akt expression in ES alone or combined with irradiation of 4Gy (Fig. 6B). Nevertheless, no significant changes of expression of phosphor-Akt were observed in irradiation-treated H-520 cells.

**Table 3:** Expressions of phospho-p38-MAPK in H-520 cells ( ± s)

	Flurescence intensity	
	
Group	Phospho-p38-MAPK(-)%	Phospho-p38-MAPK(+)%
control	87.61 ± 0.72	12.39 ± 0.72
ES	90.31 ± 0.27^a^	9.69 ± 0.27^b^
Irradiation	84.08 ± 1.39^c^	15.92 ± 1.39^d^
ES+ irradiation	88.99 ± 0.78	11.01 ± 0.78

Compared with control *t *= -6.11, ^a^*P *< 0.01; *t *= 6.11, ^b^*P *< 0.01; *t *= 3.90, ^c^*P *< 0.05; *t *= -3.90, ^d^*P *< 0.05.

## Discussion

It is known that ED inhibits the angiogenesis of malignant diseases, but it does not inhibit the functions of cancer cells. However, recent studies showed that ED possess direct antitumor activity on cancer cell migration, metastasis, and proliferation [11,17,21-23]. ES, a novel recombinant human ED, has been demonstrated to inhibit the proliferation and migration of human umbilical vein endothelial cells (HUNEC_S_) and the invasion of MDA-MB-435 human breast cancer cells [19,24]. But weather it suppress the cell proliferation of cancer cells has not been reported. In the present study, we showed the first evidence that ES directly inhibits the proliferation of human lung squamous cancer cells H-520 and induces H-520 cell apoptosis. Moreover, ES could enhance the sensitivity of H-520 cells to irradiation.

It has been reported that ED enhanced the anti-tumor effects of ionizing irradiation in vivo [25-29]. ES significantly inhibited the proliferation of H-520 cells in a dose- and time- dependent manner as determined by an MTT assay. The dose-survival curves of ES-treated H-520 cells exhibited a narrower shoulder and a greater slope rate which standard for quasi-threshold doses (D_q_) and mean lethal doses (D_0_) respectively. It indicates that the repair of sublethal damages and sensitivity to lethal radiation dose was decreased. A significant radiosensitivity effect with the sensitization enhancement ratio (SER) is 1.51. So our findings showed that ES could enhance the sensitivity of H-520 cells to irradiation and had the potential to be a radiosensitizer.

The mechanisms of ES improved H-520 cell response to radiation were multiple, especially in the in vivo models [30-32]. ES could inhibit the proliferation of endothelial cells and cancer cells, and induced cell apoptosis, both of which reduce the total tumor cell mass, making tumors more amenable to control by radiotherapy [11,18,33-35]. The cell cycle phases also determined H-520 cancer cells relative to radiosensitivity, with H-520 cells being most radiosensitive in the G_2_/M phase, less sensitive in the G_0_/G_1 _phase, and least sensitive during the latter part of the S phase [36]. It was demonstrated that ED induced a cell cycle arrest mainly in the G_0_/G_1 _phase and a decrease in the S phase [37-39]. In the current study, we observed a clear cell arrest in the G_0_/G_1 _phase and a reduction in the H-520 cells of S phase in ES-treated H-520 cells with a time-dependent style. H-520 cells progressing past the G_1_/S block might accumulate proapoptotic signals caused by both radiation and ES, resulting in increased H-520 cell death [40]. Radiation alone or the combination of radiation and ES promoted significantly higher percentages of cells in G2/M phase at 24 h after treatment, though the combination of radiation and ES showed less effect than radiation alone in this respect. These data indicate that radiation induces a cell cycle arrest in G2/M phase in H-520 cells at the early time points. However, in the later stage, more cells in this phase will process cell death so the percentage of cells in G2/M phase will decrease dramatically, as seen in 48 h after radiation.

A fundamental view on apoptosis was that apoptotic signaling transduction act mostly upon pathways initiating from mitochondria and they eventually met at the level of Bcl-2 family members and the caspases, an ultimate executioners of cell death [41]. It has been reported that ED induced apoptosis in several cancer cells [11,18], and it also has been reported that ED induced apoptosis in endothelial cells in a caspase-dependent manner, and ED-mediated apoptosis is associated with several apoptotic signaling pathways including overloading of intracellular magnesium and calcium, as well as regulation of p53 and Bcl-2 expression [10,42,43]. In the present study, we evaluated apoptosis in H-520 cells after ES combination with irradiation treatment from morphological and quantitative aspects, which showed a significant increase in H-520 cellular apoptosis. Consistently, the levels of Bcl-2 protein in H-520 cells were decreased after treatment and the level of activated caspase proteins was increased. These changes in combination treatment group were significantly greater than that in single treatment group and is consistent with the previous studies [10,42].

Previous studies showed that the PI3K-Akt pathway and MAPKs have a significant effect on the cell survival of cancer cells and play an important role in the anti-apoptotic effects of growth factors [44,45]. There are four distinct subfamilies in the MAPK family: ERKs (ERK1 and ERK2), p38 MAPK, JNK/SAPK, and ERK5/BMK1. Among them, p38 MAPK is closely related to the apoptosis of a variety of cells [46-48]. The Akt protein played a key role in a pathway related to survival by inhibition of apoptotic signals and promotion of cell cycle progression, with a clear implication in cancer and other pathologies. Also, Akt has been shown to be potently activated in response to a wide variety of growth factors and in response to DNA damage [49]. It was demonstrated that ES suppress the activation of ERKs, p38 MAPK, and Akt in HUVECs or retinal microvascular endothelial cells (RMECs) [49,50]. In the present study, ES combined with irradiation significantly reduce the phosphorylation of p38-MAPK protein and Akt protein, which probably be the mechanism leading to the increased apoptosis of H-520 after treatment.

In conclusion, ES significantly inhibited cell proliferation and induced apoptosis in human lung squamous cancer cell line H-520 and has the potential to be a radiosensitizer, which may due to the cell cycle redistribution, increase of cell apoptosis and influences on the PI3K-Akt pathway and MAPKs. The present data offer basic information of the combining ES and irradiation on tumor treatment, which may have clinical significance.

## Abbreviations

**ED**: recombinant human endostatin; **ES**: Endostar; **PE**: plating efficiency; **SF**: survival fraction; **SER**: sensitization enhancement ratio; **D**_**0**_: mean lethal dose; mean inactivation dose, **D**_**q**_: quasithreshold dose; **N**: extrapolation number; **SF**_**2**_: surviving fraction after 2Gy irradiation; **MTT**: methyl thiazolyl tetrazolium; **FCM**: flow cytometry; **NSCLC**: non-small cell lung cancer; **HUVEC**_**S**_: human umbilical vein endothelial cells.

## Competing interests

The authors declare that they have no competing interests.

## Authors' contributions

ZYY carried out cell colony-forming assay, fluorescence-activated cell sorting, flow cytometric analysis, and drafted the manuscript. JJW participated in its design and revised the manuscript. FL performed the statistical analysis. YDZ carried out the irradiation experiment. YZ supervised experimental work and revised the manuscript. All authors read and approved the final manuscript.
